# A Combined Computational Fluid Dynamics and Arterial Spin Labeling MRI Modeling Strategy to Quantify Patient-Specific Cerebral Hemodynamics in Cerebrovascular Occlusive Disease

**DOI:** 10.3389/fbioe.2021.722445

**Published:** 2021-08-17

**Authors:** Jonas Schollenberger, Nicholas H. Osborne, Luis Hernandez-Garcia, C. Alberto Figueroa

**Affiliations:** ^1^ Department of Biomedical Engineering, University of Michigan, Ann Arbor, MI, United States; ^2^ Department of Surgery, University of Michigan, Ann Arbor, MI, United States; ^3^ Functional MRI Laboratory, University of Michigan, Ann Arbor, MI, United States

**Keywords:** arterial spin labeling, computational fluid dynamics, cerebral hemodynamics, cerebrovascular occlusive disease, circle of willis, collateral flow, carotid stenosis

## Abstract

Cerebral hemodynamics in the presence of cerebrovascular occlusive disease (CVOD) are influenced by the anatomy of the intracranial arteries, the degree of stenosis, the patency of collateral pathways, and the condition of the cerebral microvasculature. Accurate characterization of cerebral hemodynamics is a challenging problem. In this work, we present a strategy to quantify cerebral hemodynamics using computational fluid dynamics (CFD) in combination with arterial spin labeling MRI (ASL). First, we calibrated patient-specific CFD outflow boundary conditions using ASL-derived flow splits in the Circle of Willis. Following, we validated the calibrated CFD model by evaluating the fractional blood supply from the main neck arteries to the vascular territories using Lagrangian particle tracking and comparing the results against vessel-selective ASL (VS-ASL). Finally, the feasibility and capability of our proposed method were demonstrated in two patients with CVOD and a healthy control subject. We showed that the calibrated CFD model accurately reproduced the fractional blood supply to the vascular territories, as obtained from VS-ASL. The two patients revealed significant differences in pressure drop over the stenosis, collateral flow, and resistance of the distal vasculature, despite similar degrees of clinical stenosis severity. Our results demonstrated the advantages of a patient-specific CFD analysis for assessing the hemodynamic impact of stenosis.

## Introduction

Cerebrovascular occlusive disease (CVOD), characterized by the presence of stenosis in the arteries supplying the brain, is a major risk factor for ischemic stroke. Clinical diagnosis and stratification of CVOD patients relies routinely on measuring the maximum narrowing of the lumen based on duplex ultrasound or computed tomography angiography (CTA). However, the degree of luminal stenosis is only one factor in the assessment of stroke risk. Plaque characteristics, downstream brain perfusion, and patency of collateral pathways also play an important role in the overall risk evaluation of cerebral ischemia (Saba et al., 2018; Liebeskind, 2003; Liebeskind and Feldmann, 2012). Collateral flow in the circle of Willis (CoW) has been associated with reduced stroke risk in patients with severe carotid stenosis (Henderson et al., 2000; Bisschops et al., 2003; Hendrikse et al., 2001). Collateral flow is highly dependent on the cerebral vasculature anatomy, availability of collateral pathways, degree of stenosis in the arteries supplying the brain and, critically, the condition of the cerebral microcirculation and its autoregulatory response (Ramsay et al., 1991; Russell et al., 2008).

The clinical gold standard for evaluating collateral flow is digital subtraction angiography (DSA). Despite providing high-resolution images of blood supply in the cerebral arteries, the procedure is invasive and strictly qualitative. MRI arterial spin labeling (ASL) has become an increasingly popular method for measuring cerebral perfusion, and it provides a non-invasive quantitative alternative to DSA. In non-selective ASL (NS-ASL), brain tissue perfusion is measured by magnetically labeling blood in the neck arteries and acquiring a series of slices of the brain after a short transit delay (Alsop et al., 2014). More recently, ASL has been extended to vessel-selective labeling to measure the perfusion territory of individual arteries (Helle et al., 2010; Schollenberger et al., 2020). The diagnostic capabilities of vessel-selective ASL (VS-ASL) have previously been demonstrated in patients with extracranial stenosis and arteriovenous malformation (Richter et al., 2017; Helle et al., 2013). Additionally, cerebral angiograms have been performed based on VS-ASL to visualize blood supply in the cerebral arteries (Jensen-Kondering et al., 2015), rendering similar qualitative information on cerebral flow patterns as DSA. Nevertheless, the information provided by VS-ASL on collateral flow patterns has thus far been qualitative.

Image-based computational fluid dynamics (CFD) provides a powerful tool for analyzing cerebral hemodynamics. Compared to experimental approaches, CFD renders velocity, pressure, wall shear stress, etc. throughout entire vascular territories with arbitrarily high spatial and temporal resolutions. The feasibility of CFD to assess cerebral hemodynamics has been previously demonstrated for intracranial stenoses (Liu et al., 2017; Leng et al., 2014) and aneurysms (Castro et al., 2006; Rayz et al., 2008; Raschi et al., 2012). However, patient-specific calibration of cerebral blood flow CFD models remains challenging. Previous studies have heavily relied on literature data for determining flow splits in the CoW (Xiao et al., 2013; Mukherjee et al., 2016) or used simplistic allometric scaling assumptions to calibrate outflow boundary conditions (Bockman et al., 2012).

In this paper, we propose a novel strategy to quantitatively characterize regional cerebral blood flow and perfusion using CFD in combination with PC-MRI and ASL data. First, a method to calibrate the cerebral blood flow CFD model based on NS-ASL perfusion data is presented. The calibration includes estimation of flow splits in the CoW from non-selective perfusion images and total inflow to the CoW from PC-MRI, followed by tuning of the outflow boundary conditions to match the estimated flow splits. Second, the calibrated CFD model is validated against territorial perfusion maps from VS-ASL based on the blood supply to each cerebral territory using Lagrangian particle tracking (LPT). Lastly, the proposed strategy is demonstrated *via* an in-depth quantification of patient-specific cerebral hemodynamics in a healthy control subject and two CVOD patients.

## Materials and Methods

### Patient Details

**FIGURE 1:**
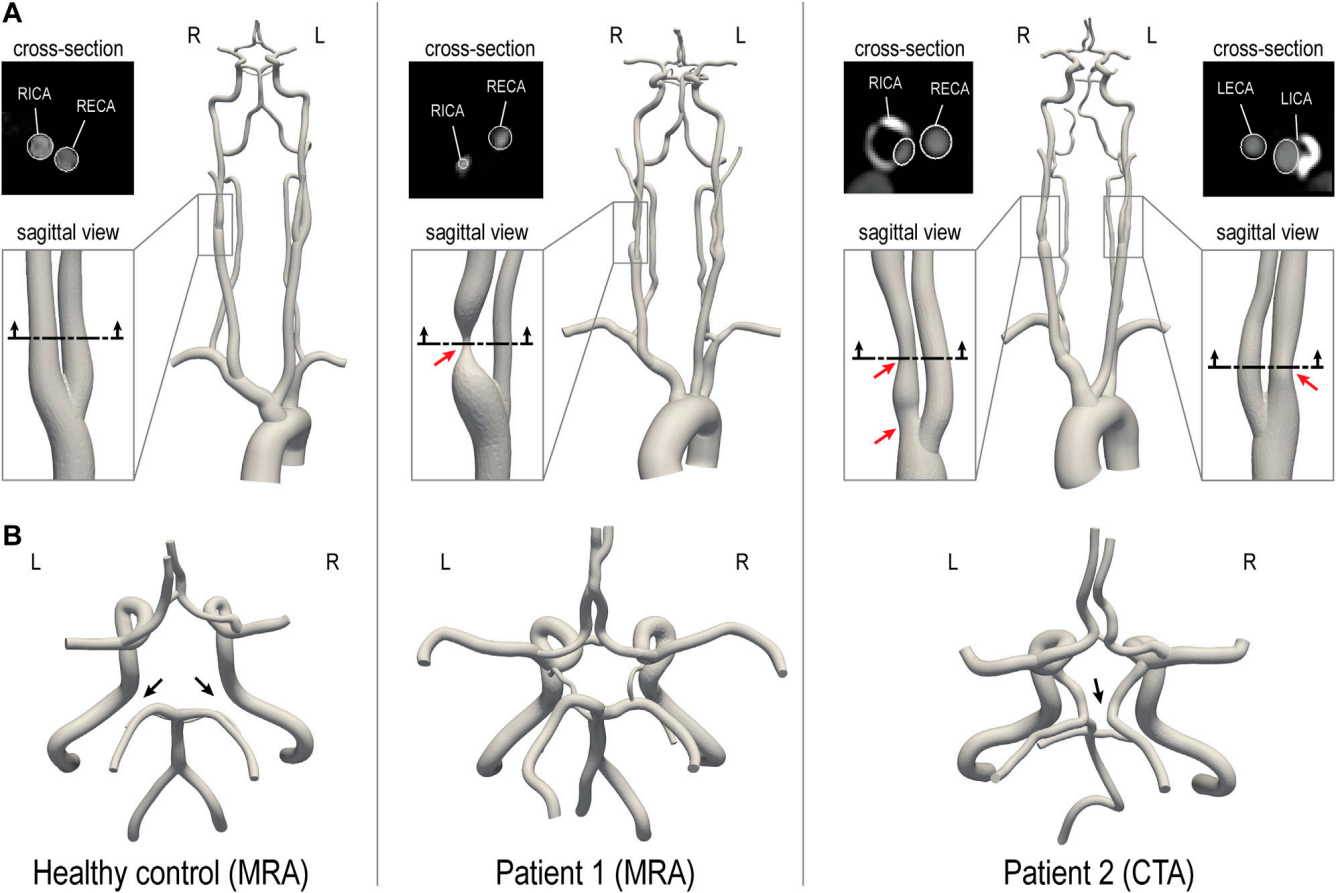
3D-reconstructed geometric models of a healthy control and two CVOD patients. **(A)** For each patient, a close-up of the stenosis is shown. The red arrows indicate the location of the stenosis. An axial cross-section of the stenosis illustrates a comparison between image data and model contours (this comparison is also shown for the healthy volunteer). **(B)** Posterior view of the CoW. The black arrows indicate variations in the CoW anatomy. RICA, right internal carotid artery; RECA, right external carotid artery; LICA, left internal carotid artery; LECA, left external carotid artery. R and L indicate the right and left side from the subject’s perspective.

Two CVOD patients and a healthy control subject were enrolled in a feasibility study and underwent a research MRI exam. The protocol was approved by the local Institutional Review Board and all subjects provided informed written consent (HUM00114275 and HUM00018426). The reconstructed geometric models of the three subjects are illustrated in Figure 1. The models include the ascending and proximal descending thoracic aorta, its upper branches (brachiocephalic trunk, left carotid and left subclavian), the main neck arteries (internal and external carotids, vertebral arteries), and the main intracranial arteries including the CoW. The healthy control and patient 1 were reconstructed based on magnetic resonance angiography (MRA) and patient 2 based on CTA.

The healthy control subject (male, 28 years old) presented without evidence of CVOD. The CoW anatomy was incomplete with both right and left posterior communicating arteries hypoplasia. Patient 1 (female, 55 years old) presented with an asymptomatic 70–99% stenosis (duplex ultrasound, velocity criteria) in the right proximal internal carotid artery (RICA). The left internal carotid artery (LICA) was patent with no evidence of hemodynamically significant stenosis. Patient 1 has a complete CoW anatomy. Patient 2 (male, 64 years old) presented with asymptomatic bilateral carotid stenosis. The RICA revealed a tandem stenosis of 80–90% (CTA, ECST criteria), stretching from the carotid bifurcation to the distal end of the carotid bulb. The LICA showed a 60% stenosis (CTA, ECST criteria). Patient 2 has an incomplete CoW anatomy with right P1 segment and distal right vertebral artery (RVA) hypoplasia.

### Imaging Data

All subjects underwent an MRI study to collect data on vascular anatomy, brain tissue perfusion, and flow. The protocol was performed at 3T (MR750; GE Healthcare, Waukesha, WI) with a 32-channel receive-only head coil for the head and neck and the scanner’s build-in coil for the upper chest. At the end of the study, the subject’s blood pressure was measured in the upper arm in the supine position.

#### Anatomy

Anatomical information from the ascending thoracic aorta to the carotid bifurcation level was acquired with a 2D T1-weighted spoiled gradient echo sequence (voxel size = 0.58 × 0.58 × 2 mm^3^, TR/TE = 75/3.3 ms). The remaining anatomy from the neck to the CoW was acquired with a 3D Time-of-Flight sequence (voxel size = 0.42 × 0.42 × 1.5 mm^3^, TR/TE = 21/2.5 ms). Additionally, structural images of the brain were collected with a 2D T1-weighted spoiled gradient echo sequence (voxel size = 0.46 × 0.46 × 7 mm^3^, TR/TE = 100/3.0 ms). For patient 2, an additional CTA data of the neck and head vasculature was available (voxel size = 0.39 × 0.39 × 0.62 mm^3^). For patient 2, the CTA dataset was chosen over the MRA for reconstruction due to the higher resolution.

#### Brain Tissue Perfusion

Using a pseudo-continuous ASL scheme, non-selective and vessel-selective cerebral perfusion images were collected. Prior to image acquisition, an off-resonance calibration pre-scan was performed to correct for B0-inhomogeneity in the label plane. For the NS-ASL acquisition, sequence parameters were set following consensus recommendations (Alsop et al., 2014): Label duration = 1,800 ms, post-labeling delay = 2,000 ms, TR/TE = 4,600/4, voxel size = 3.75 × 3.75 × 7 mm^3^, 3D spiral acquisition, 18 slices, 8 pairs of label/control images. The slice prescription was the same as for the T1-weighted structural images. The label plane was positioned above the carotid bifurcation where the arteries of interest (carotid and vertebral) run perpendicular to the plane and with a maximum distance between them. The start of the labeling period was cardiac-triggered to reduce pulsatility artifacts. A proton density image was collected, followed by a non-selective perfusion scan. Subsequently, four VS-ASL scans of the vertebral and carotid arteries were collected. The position of vertebral and carotid arteries within the label plane was determined from the Time-of-Flight acquisition. Keeping all parameters of the NS-ASL scan unchanged, vessel-selective labeling was performed based on a super-selective labeling scheme (Helle et al., 2010), whereby additional in-plane gradients rotate clockwise every radiofrequency pulse to create a circular labeling spot. Finally, the vessel-selective labeling efficiency was measured by collecting an image 2 cm above the labeling plane 10 ms after labeling for 500 ms. Image reconstruction was performed in MATLAB to a resolution of 128 × 128 using zero-padding in k-space. The ASL perfusion signal was calculated by subtracting label and control images and averaging over all acquired pairs. A detailed explanation of the sequence setup and parameters can be found elsewhere (Schollenberger et al., 2020).

#### Flow

Volumetric blood flow waveforms were measured using 2D cardiac-gated phase-contrast (PC-MRI) at the level of the ascending aorta (voxel size = 0.58 × 0.58 × 5 mm^3^, TR/TE = 5.2/3.1 ms, velocity encoding = 130 cm/s) and above the carotid bifurcation (voxel size = 0.31 × 0.31 × 5 mm^3^, TR/TE = 6.0/3.7 ms, velocity encoding = 100 cm/s). The slice was positioned perpendicular to the arteries of interest and velocity was encoded in the through-plane direction. PC-MRI data were processed in MATLAB to calculate flow rates.

### Computational Modeling

The key computational modeling tasks, namely three-dimensional anatomical reconstruction, mesh generation, boundary condition specification, and finite element analysis were performed using the validated open-source computational hemodynamics framework CRIMSON (Arthurs et al., 2021).

#### Anatomical Reconstruction and Mesh Generation

3D geometric models of the aorta and head and neck vessels, including the CoW, were reconstructed from the anatomical imaging data. Briefly, centerlines and 2D vessel contours were defined for each vessel of interest. Contours were then lofted to create an analytical representation of each vessel and ultimately define a 3D geometric model of the vasculature (Xiao et al., 2013). This 3D model was then discretized using linear tetrahedral elements. A mesh-adaptation algorithm (Sahni et al., 2006) was used to refine the mesh locally based on local velocity gradients. The final mesh sizes for healthy subject, patient 1, and patient 2 consisted of 2.16 × 10^6^, 1.84 × 10^6^, and 2.39 × 10^6^ elements, respectively. Mesh-independence was evaluated for patient 1 by creating an additional highly-refined mesh with 6.86 × 10^6^ elements, which resulted in a difference of less than 1% for the flow rates at each outlet and a maximum difference of 2% for peak velocity in the center of the stenosis.

#### Boundary Conditions

A pulsatile inflow waveform reconstructed from PC-MRI was mapped to a parabolic velocity profile and prescribed at the inlet of the ascending aorta for each geometric model. Each vessel outlet was coupled to a three-element Windkessel model, which consists of a proximal resistance (R_p_), a distal resistance (R_d_), and a capacitor (C) (Vignon-Clementel et al., 2010). For each subject, the total arterial resistance is R_T_ = P_mean_/Q_T_, where the mean pressure P_mean_ = 1/3 P_systolic_ + 2/3 P_diastolic_ and Q_T_ is total cardiac output. The total arterial compliance is C_T_ = (Q_T,max_−Q_T,min_)/(P_systolic_−P_diastolic_)*∆t, where Q_T,max_ and Q_T,min_ are maximum and minimum values of aortic inflow, and ∆t is the time lapse between these values. Initial estimates for the Windkessel model parameters were obtained by distributing R_T_ and C_T_ among the different outlets, to obtain R_i_ and C_i_ for vessel i = 1,…,13, as described in (Xiao et al., 2014). The Windkessel parameters were then iteratively adjusted following the scheme described in detail in *Patient-specific calibration of outflow boundary conditions for the CFD models*. Lastly, a no-slip boundary condition was assigned to all vessel walls.

#### Finite Element Analysis

Blood was modeled as an incompressible Newtonian fluid with a dynamic viscosity of 0.004 kg m^−1^s^−1^ and a density of 1,060 kg m^−3^. A stabilized finite-element formulation for the incompressible Navier-Stokes equations was employed to solve for blood flow velocity and pressure in the models (Whiting and Jansen, 2001). Computations were performed using 80 cores on a high-performance computing cluster. Simulations were run using a time step size of 0.1 ms until cycle-to-cycle periodicity was achieved, typically after 5 cardiac cycles.

### Patient-Specific Calibration of Outflow Boundary Conditions for the CFD Models

#### Calculation of Mean Flow at Model Outlets

##### Intracranial Arteries

**FIGURE 2:**
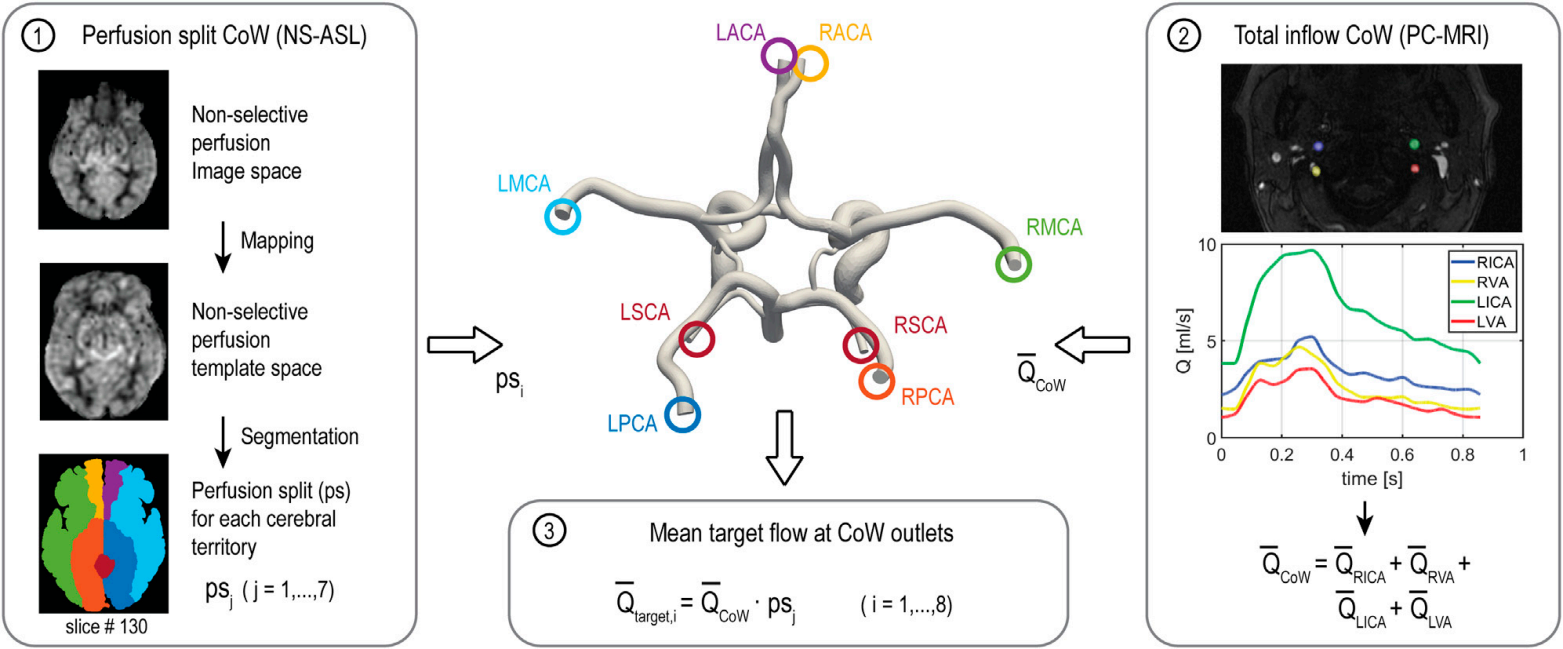
Workflow of calculating the flow split among the arteries of the CoW based on NS-ASL perfusion imaging, a vascular territory atlas, and total inflow to the CoW from PC-MRI. RACA/LACA, right/left anterior cerebral artery; RMCA/LMCA, right/left middle cerebral artery; RPCA/LPCA, right/left posterior cerebral artery; RSCA/LSCA, right/left superior cerebellar artery.

The flow distribution among cerebral and cerebellar arteries was derived from the NS-ASL perfusion images, a cerebral territory atlas, and the total inflow to the brain as illustrated in Figure 2. First, the NS-ASL perfusion images were mapped into a standardized template space (Montreal Neurological Institute ch2better) using the toolbox SPM12 (Wellcome Trust Center for Neuroimaging, London, United Kingdom). Next, the standardized perfusion images were segmented using a vascular territory atlas. The atlas was extended to include the cerebellum. This extended vascular territory atlas with a resolution of 370 × 301 × 316 is given as a NIfTI dataset in the Supplementary Materials. In this work, we assumed the following relationship between eight intracranial arteries and seven vascular territories (see Figure 2): 1) RACA territory, perfused by the RACA (yellow); 2) LACA territory, perfused by the LACA (magenta); 3) RMCA territory, perfused by the RMCA (green); 4) LMCA territory, perfused by the LMCA (light blue); 5) RPCA territory, perfused by the RPCA (orange); 6) LPCA territory, perfused by the LPCA (dark blue); 7) Cerebellum territory, perfused evenly by RSCA and LSCA (red). A perfusion split ps_j_ was then calculated by dividing the integral of perfusion signal over the volume of each territory j = 1,…,7 by the integral of perfusion signal over the entire brain volume. The total mean inflow to the CoW (Q̅_CoW_) was calculated from the PC-MRI data on left and right ICAs and VAs. Mean flow rates through each intracranial vessel (Q̅_target_,_i_) were calculated as the product of the total mean inflow to the CoW and the perfusion split ps_j_ corresponding to the territory perfused by vessel i = 1,…,8.

##### Extracranial Arteries

Mean flow rates in the external carotid arteries were calculated from PC-MRI. In the subclavian arteries, we assumed a mean flow rate of 5.6% of cardiac-output (Xiao et al., 2013). Finally, the difference between inflow and intracranial, external carotid arteries, and subclavian arteries flow was assigned to the descending thoracic aorta.

#### Calibration of Windkessel Model Parameters

Patient-specific calibration of the Windkessel model parameters for each outflow branch was performed in three stages. *Stage 1*: the distal resistance R_d_ was iteratively adjusted during simulation runtime using Python controller scripts (Arthurs et al., 2016) to match the target mean flow rates. At each simulation time step, R_d_ was adjusted proportional to the error between the current mean flow and the target flow. Simulations were terminated once the flow at each outlet was fully converged (error <1%). *Stage 2:* The ratio of R_p_/R_d_ was adjusted for each cerebral and cerebellar branch such that the computed and measured PC-MRI flow waveforms in the ICAs and VAs had similar pulsatility. The total resistance R_i_ = R_p_ + R_d_ at each outlet was kept constant to preserve mean flow. *Stage 3:* Measurements of brachial pressure (P_systolic_ and P_diastolic_) were matched by adjusting R_T_ and C_T_. The percentage change in R_T_ and C_T_ through the iterations was proportionally assigned to R_i_ and C_i_ at each outlet i = 1,…,13. The final Windkessel parameters are summarized in Supplementary Table S1.

### Validation of the Calibrated CFD Models

The calibrated CFD models were validated against VS-ASL by comparing the fractional blood supply (FBS) in each vascular territory. We defined the fractional blood supply for a vascular territory *j* from a neck artery *k* as FBS_j,k_ = Q_j,k_/Ʃ_k_ Q_j,k_, where *j =* 1,…,7 is the vascular territory index and *k =* 1,…,4 is the neck artery index (1:RIVA, 2:RVA, 3:LVA, 4:LICA), and Q_j,k_ is the flow contribution from the neck artery *k* to the vascular territory *j*. The process for calculating FBS_j,k_ from VS-ASL and CFD data is described next.

#### Fractional Blood Supply Based on VS-ASL

**FIGURE 3:**
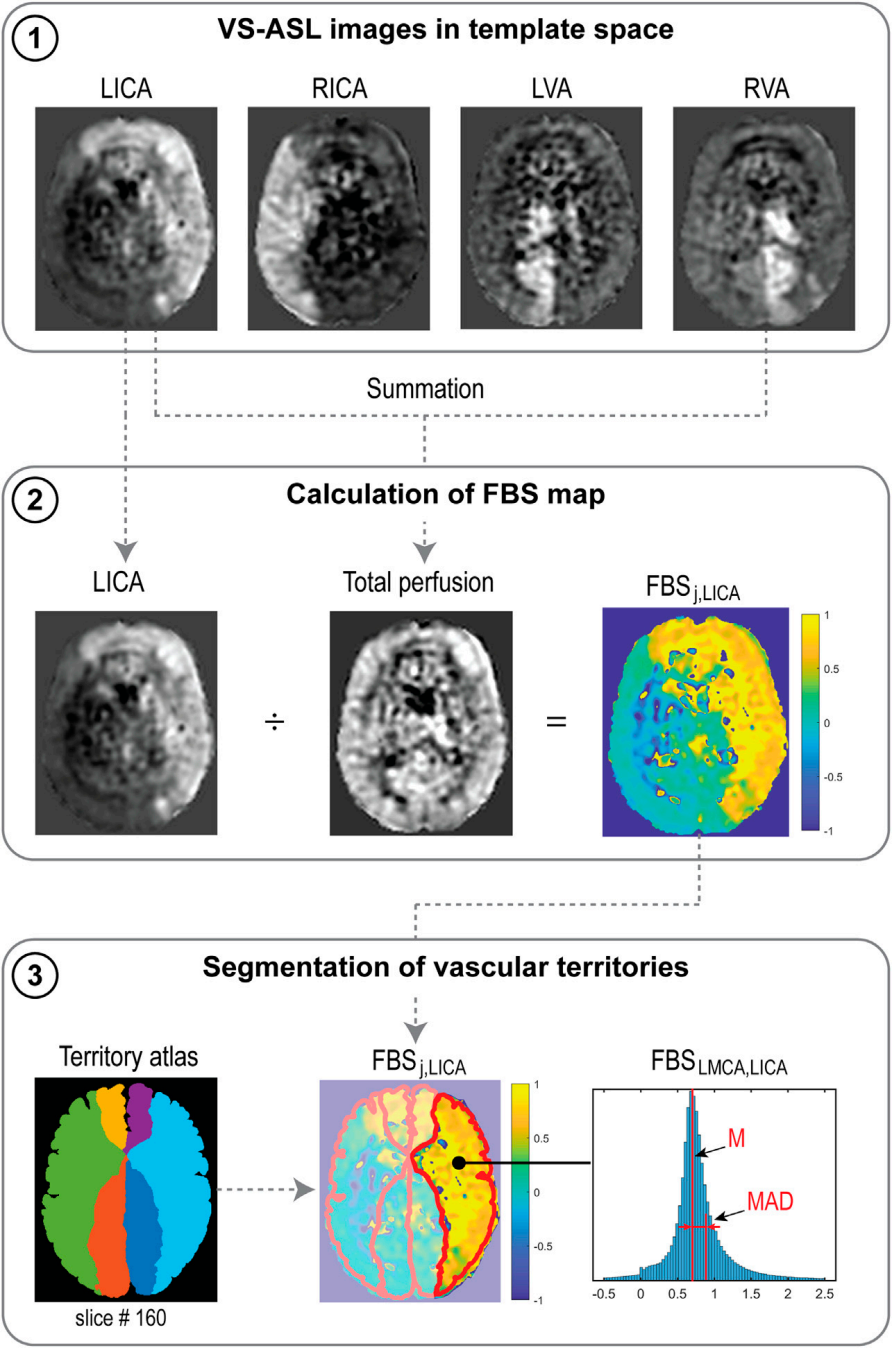
Processes to calculate FBS_j,k_ from VS-ASL images, illustrated for the LICA. (1) Scaled VS-ASL perfusion images in standardized template space. (2) Calculation of spatial FBS_j,k_ maps. (3) Segmentation of territories using a vascular territory atlas and calculation of median (M) and median absolute deviation (MAD) from the FBS_j,k_ distribution in each territory.

The process for calculating FBS_j,k_ from VS-ASL images is outlined in Figure 3. The perfusion signal in the VS-ASL images is determined by the blood supply from a single neck artery to the vascular territories. The signal in the VS-ASL images was first scaled based on the measured labeling efficiency. Then, scaled VS-ASL images were transformed into the standardized template space (panel 1). The sum of all scaled VS-ASL images produces the total perfusion image. The FBS_j,k_ maps were then calculated by dividing each scaled VS-ASL image by the total perfusion image on a voxel-by-voxel basis (panel 2). Then, the FBS_j,k_ maps were segmented into different territories using the vascular territory atlas. Lastly, due to noise in the raw ASL data, negative values of FBS_j,k_ distribution are possible on a given voxel. Therefore, we characterize the FBS_j,k_ distribution by its median (M) and median absolute deviation (MAD) (see panel 3).

#### Fractional Blood Supply Based on CFD Lagrangian Particle Tracking

To validate the CFD results using the VS-ASL data, one must develop a method to assess the fractional blood supply of the intracranial arteries/territories and compare it with the median (M) of the FBS_j,k_ obtained with VS-ASL for each artery/territory. This can be achieved *via* a further post-processing analysis on the CFD data, known as “Lagrangian particle tracking” (LPT), whereby virtual boluses of blood are created by seeding mass-less particles in different regions of the vasculature and advected by the velocity field over multiple cardiac cycles. This is an established technique with multiple applications in cardiovascular flows (Di Achille et al., 2014; Van Bakel et al., 2018; Nauta et al., 2017; Suh et al., 2011; Arzani et al., 2014).

In this work, particles were seeded continuously at the base of the carotid and vertebral arteries. LPT was performed individually for each carotid and vertebral artery over 4 cardiac cycles (Supplementary Figure S1). The number of particles seeded in each artery, over multiple re-injections over the 4 cardiac cycles, was proportional to the flow rate of each artery. Particles were then counted at each outlet of the intracranial arteries, and once cycle-to-cycle periodicity in the number of tracked particles was achieved, the total number of particles collected per vessel over a full cardiac cycle was extracted. Lastly, for each intracranial vessel, assigned to a vascular territory *j*, its fractional blood supply FBS_j,k_ from each neck artery *k* was calculated by dividing the particle count of the LPT analysis for neck artery *k* by the sum of the particle counts of each of the four LPT analyses of the neck arteries.

## Results

### Validation of Calibrated CFD Model

#### Fractional Blood Supply: CFD LPT Versus VS-ASL

##### Qualitative Analysis

**FIGURE 4:**
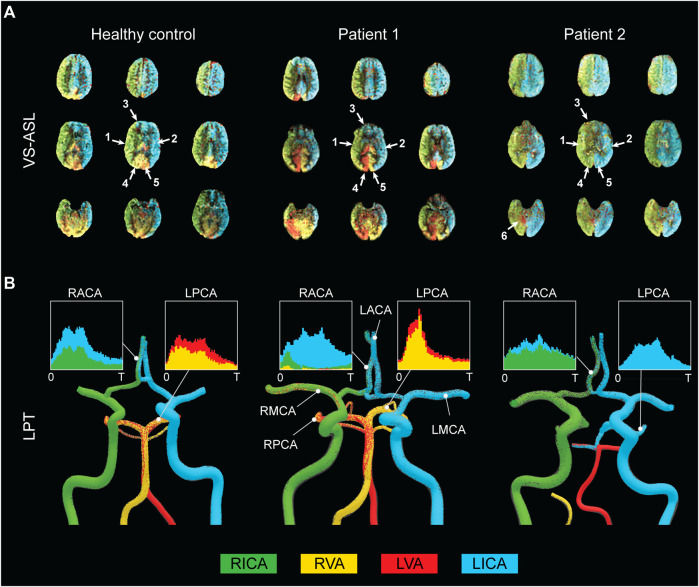
Qualitative comparison between FBS obtained from VS-ASL and CFD LPT **(A)** VS-ASL images show the perfusion territories of the main neck arteries from the inferior of the cranium **(bottom row slices)** to the superior **(top row slices)**. The images were created by color-coding the FBS maps of the main neck arteries on a voxel-by-voxel basis. For visualization purposes, we limited the fractional contributions of each neck artery to a positive range between 0 and 1. The arrows indicate the vascular territories of the 1) RMCA, 2) LMCA, 3) RACA, 4) RPCA, 5) LPCA, and 6) cerebellum. **(B)** LPT analyses show the advection of particles in the large arteries of the CoW. Particles are color-coded based on the artery of origin in the neck. Histograms demonstrate mixed supply in the RACA and LPCA over the cardiac cycle T.

A qualitative comparison between FBS obtained from VS-ASL and CFD LPT is illustrated in Figure 4. The VS-ASL images (Figure 4A) show the perfusion territories of the four neck arteries, from the inferior region of the cranium (bottom row slices) to the superior region (top row slices). Each image voxel was color-coded based on the FBS of the neck arteries. For visualization purposes, we limited the FBS in each voxel to positive fraction values between 0 and 1 (cf. Figure 3 panel 3). LPT analyses results (Figure 4B) show maps of the advection of particles, color-coded based on the seeding artery in the neck, as well as temporal histograms of particles collected at the outflow of selected intracranial arteries.

1. Middle cerebral arteries: The VS-ASL data revealed that the perfusion territories of the RMCA (arrow 1) and LMCA (arrow 2) were primarily supplied by the ipsilateral carotid artery for all subjects regardless of the degree of stenosis. This perfusion pattern was also replicated in the LPT analyses, where particles exiting the RMCA primarily originated in the RICA (green particles) and particles leaving the LMCA predominantly originated in the LICA (blue particles).
2. Anterior cerebral arteries: All three subjects displayed flow compensation from the LICA to the RACA territory (arrow 3) in the VS-ASL images. The LPT analysis also reproduced this flow compensation in the RACA *via* the anterior communicating artery (AComA) for all subjects. The histograms of LPT RACA particles illustrate clear differences in the amount of compensation among subjects. The healthy control subject revealed significant blood supply from the LICA (blue particles) to the RACA, despite the absence of stenosis. The severe RICA stenosis in patient 1 resulted in the RACA being predominantly supplied by the LICA. In contrast, the severe stenosis in the RICA and the mild stenosis in the LICA in patient 2 only led to a small amount of collateral flow in the RACA.
3. Posterior cerebral arteries: The VS-ASL images revealed large differences in blood supply in the RPCA (arrow 4) and LPCA (arrow 5) territories between subjects. These differences in blood supply were also replicated in the LPT analyses. In the healthy control subject, the posterior circulation received mixed supply from both vertebral arteries as seen in the LPCA histogram. In patient 1, the RPCA territory was predominantly perfused by the LVA whereas the LPCA territory was predominantly perfused by the RVA (see histogram). The VS-ASL data revealed a switch in blood supply to the posterior circulation between right and left hemisphere, a switch also mirrored in the LPT analysis which shows vortex-like flow patterns in the basilar artery resulting in crossing of particles originating in the VAs. In patient 2, the posterior circulation was supplied by the ipsilateral carotid arteries. In this patient, the VAs did not contribute to cerebral blood flow. Instead, the LVA supplied most of the cerebellum flow with some small contribution from the LICA, as also apparent in the VS-ASL data (arrow 6).

##### Quantitative Analysis

**FIGURE 5:**
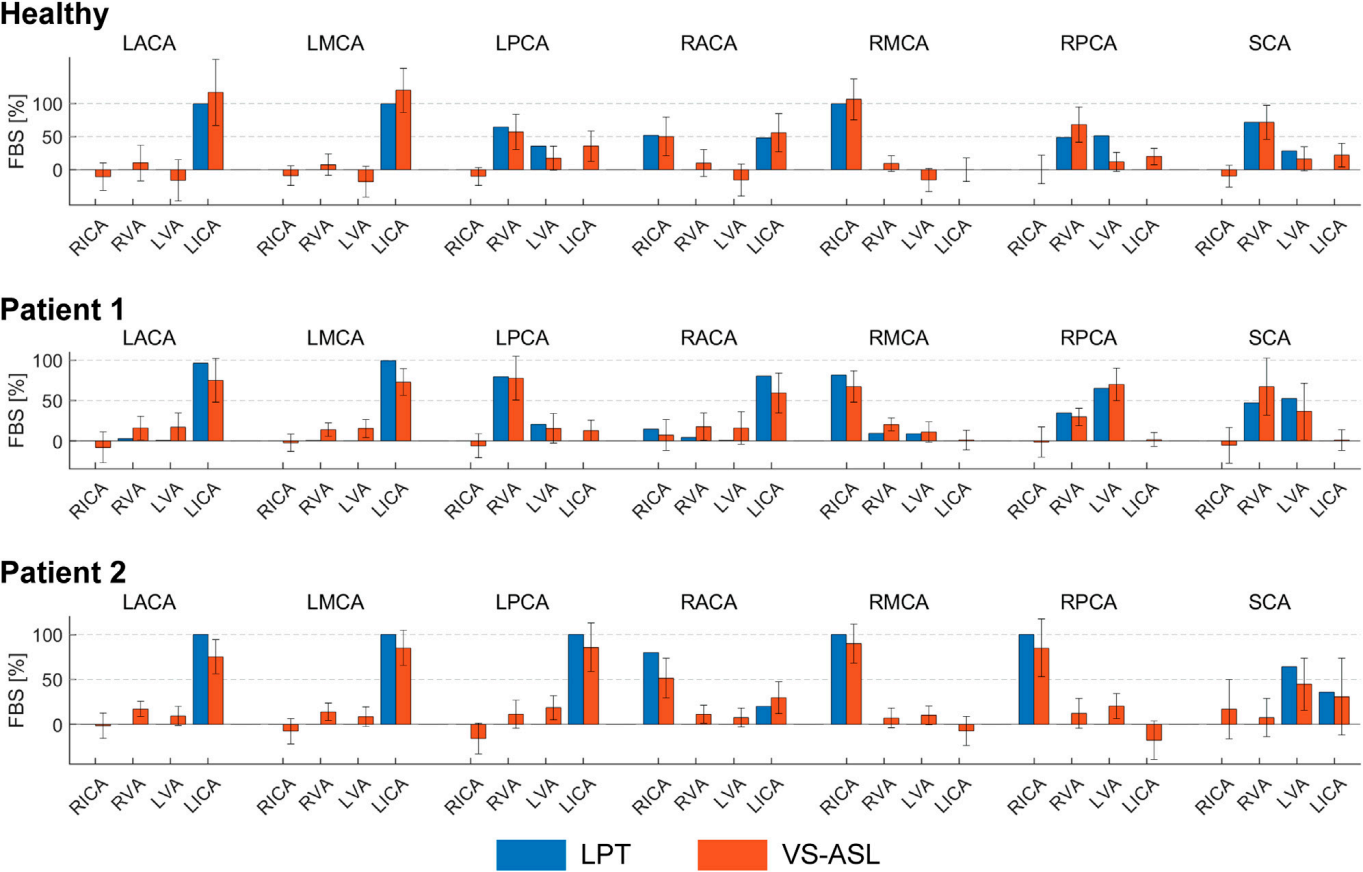
Comparison of the FBS_j,k_, obtained from VS-ASL and LPT, in each vascular territory *j* and for each neck artery *k*. For VS-ASL, values of FBS_j,k_ represent the median of the FBS_j,k_ distribution in each vascular territory. The error bar represents the median absolute deviation. For LPT, values of FBS_j,k_ were calculated based on the particle count at each outlet of the CoW. RACA/LACA, right/left anterior cerebral artery; RMCA/LMCA, right/left middle cerebral artery; RPCA/LPCA, right/left posterior cerebral artery; SCA, superior cerebellar arteries; RVA/LVA, right/left vertebral artery; RICA/LICA, right/left internal carotid artery.

A quantitative comparison of FBS_j,k_ obtained with VS-ASL and CFD LPT is summarized in Figure 5. For each vascular territory *j*, the percentage supply contributions from the neck arteries *k,* obtained from VS-ASL (red bars) and LPT (blue bars), are shown. VS-ASL data includes the median absolute deviation. Due to noise in the VS-ASL signal, FBS results show small negative values in territories for which the perfusion contribution from a given neck artery *k* is small. Conversely, the LPT data is “noise-free” and given by a single value of FBS, instead of by a distribution. Overall, VS-ASL and LPT estimates of FBS_j,k_ agreed well for all subjects. The LPT analysis correctly identified the artery contributing the largest % of perfusion in vascular territories predominately perfused by a single neck artery in all subjects (e.g., LACA, LMCA, RMCA). Furthermore, the magnitude of flow compensation from the LICA to the RACA territory was correctly reflected in the LPT analyses for all subjects. The main sources of perfusion in the RPCA and LPCA territories were correctly identified by the LPT in both patients and only partially matched in the healthy control subject.

**FIGURE 6:**
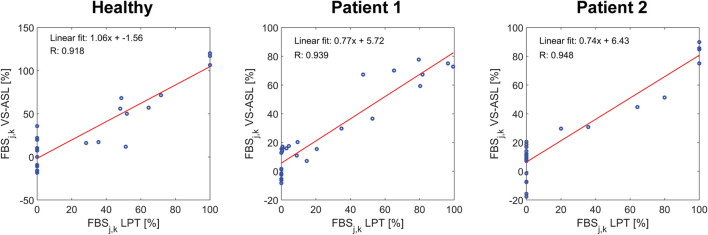
Correlation of FBS_j,k_ between VS-ASL and LPT. For each subject, the correlation coefficient and linear fit of FBS_j,k_ over all territories *j* and neck arteries *k* was calculated.

A correlation coefficient of FBS_j,k_ between VS-ASL and LPT was calculated for each subject over all vascular territories *j* and neck arteries *k* (Figure 6). The correlation coefficients were R = 0.92, R = 0.94, and R = 0.95 for the healthy subject, patient 1, and patient 2, respectively.

#### Flow: CFD Versus PC-MRI

**FIGURE 7:**
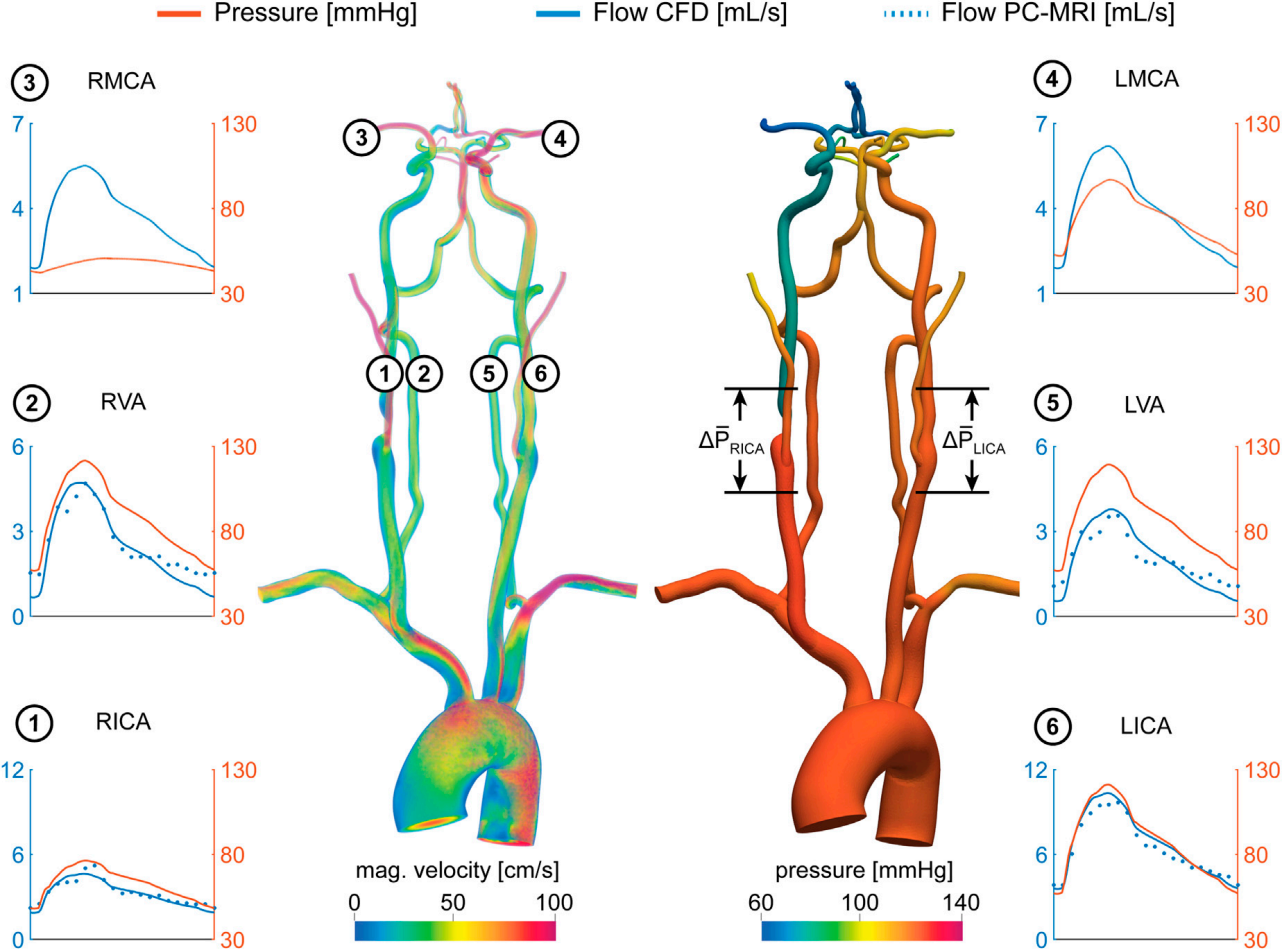
Velocity and pressure fields at peak systole for patient 1. Flow and pressure waveforms are evaluated in the internal carotid (ICA), vertebral (VA), and middle cerebral arteries (MCA). The flow waveforms in the neck arteries are compared to PC-MRI measurements above the carotid bifurcation (dotted lines). The mean pressure drop ∆P̅ = P̅_prox_−P̅_dist_ was calculated over the RICA stenosis and the same vessel segment in the LICA.

**FIGURE 8:**
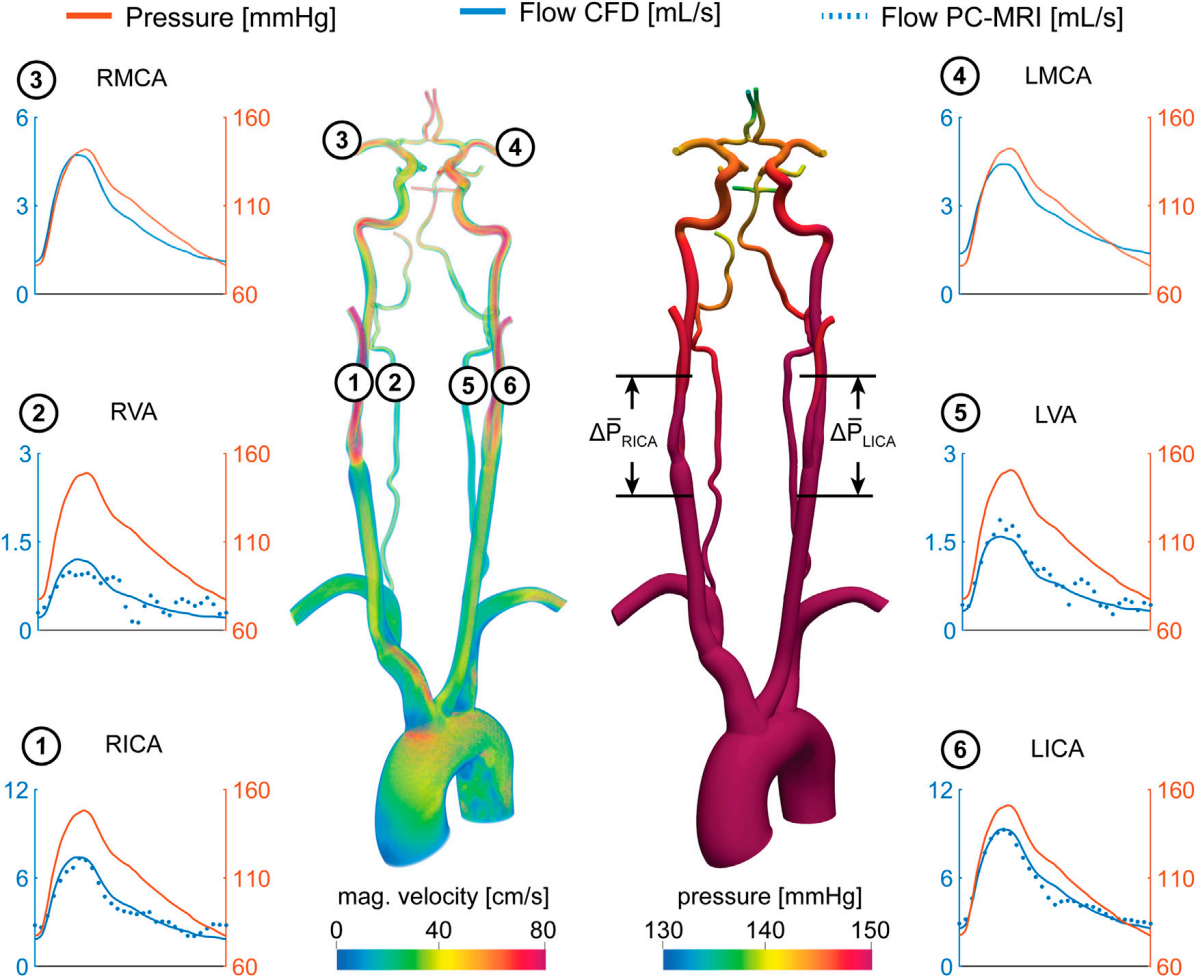
Velocity and pressure fields at peak systole for patient 2. Flow and pressure waveforms are evaluated in the internal carotid (ICA), vertebral (VA), and middle cerebral arteries (MCA). The flow waveforms in the neck arteries are compared to PC-MRI measurements above the carotid bifurcation (dotted lines). The mean pressure drop ∆P̅ = P̅_prox_−P̅_dist_ was calculated over the RICA and LICA stenosis.

Mean flow rates from the calibrated CFD model were compared to PC-MRI flow data in the vertebral and carotid arteries above the carotid bifurcation. The difference in mean flow rates in each neck artery was smaller than 10% for all subjects. A comparison of the flow waveforms in the vertebral and carotid arteries between CFD and PC-MRI is shown in Figure 7 for patient 1, Figure 8 for patient 2, and Supplementary Figure S2 for the healthy control subject.

### CFD-Based Quantification of Patient-specific Cerebral Hemodynamics

Once the validation of the CFD results using VS-ASL and PC-MRI data was established, we used the calibrated models to assess alterations in cerebral hemodynamics. Specifically, pressure and flow waveforms, the hemodynamic impact of the carotid stenoses, and the resistances of the distal cerebral vascular territories were quantified in the two patients of the study.

Figure 7 shows pressure and flow waveforms in six arteries of the neck and head for patient 1. The mean pressure drop over the stenosis was defined as ∆$\bar{P}$ = $\bar{P}$_prox_-$\bar{P}$_dist_, where $\bar{P}$_prox_ and $\bar{P}$_dist_ are the mean cross-sectional-averaged pressures 2 cm proximal and distal to the maximum diameter reduction, respectively. The mean pressure drop over the RICA was ∆$\bar{P}$_RICA_ = 26.25 mmHg, compared to a ∆$\bar{P}$_LICA_ = 0.58 mmHg mean pressure drop over the unstenosed LICA. Another metric of the hemodynamic significance of the stenosis is given by the fractional flow (FF) index (Liebeskind and Feldmann, 2012; Liu et al., 2017; Miao et al., 2016), defined as FF = $\bar{P}$_dist_ $/\bar{P}$_prox_. This index produced FF_RICA_ = 0.71 and FF_LICA_ = 0.99. The stenosis resulted in a substantial difference in flow between the RICA and LICA, with mean flow rates of $\bar{Q}$_RICA_ = 3.25 ml/s and $\bar{Q}$_LICA_ = 6.79 ml/s. In the CoW, the RMCA and LMCA exhibit substantially different mean values of pressure ($\bar{P}$_RMCA_ = 47.62 mmHg, $\bar{P}$_LMCA_ = 74.91 mmHg, mean difference: 27.29 mmHg). Despite the pressure difference between hemispheres, the mean flow rates in the RMCA and LMCA were comparable with $\bar{Q}$_RMCA_ = 3.74 ml/s and $\bar{Q}$_LMCA_ = 3.92 ml/s. This preservation of flow at the RMCA was achieved through a reduction in the total resistance of its distal vasculature, resulting in R_RMCA_ = 1.7 × 10^9^ Pa s m^−3^ compared to R_LMCA_ = 2.54 × 10^9^ Pa s m^−3^ at the contralateral LMCA.

Figure 8 shows the pressure and flow waveforms for patient 2. The mean pressure drop over the stenoses and *FF* indices in the RICA and LICA were ∆$\bar{P}$_RICA_ = 1.75 mmHg, ∆$\bar{P}$_LICA_ = 0.71 mmHg, FF_RICA_ = 0.98, and FF_LICA_ = 0.99, respectively. The mean flow rates in the RICA and LICA were $\bar{Q}$_RICA_ = 4.00 ml/s and $\bar{Q}$_LICA_ = 5.25 ml/s. Despite the severe RICA stenosis and moderate LICA stenosis, the mean pressure at the RMCA and LMCA were similar ($\bar{P}$_RMCA_ = 106.42 mmHg, $\bar{P}$_LMCA_ = 106.37 mmHg, mean difference: 0.05 mmHg). The mean flow rates, as well as the corresponding total resistances, at the outlets of the RMCA and LMCA were comparable with $\bar{Q}$_RMCA_ = 2.50 ml/s and $\bar{Q}$_LMCA_ = 2.60 ml/s, and R_RMCA_ = 5.64 × 10^9^ Pa s m^−3^ and R_LMCA_ = 5.44 × 10^9^ Pa s m^−3^, respectively.

Pressure and flow waveforms for the healthy control subject are provided in Supplementary Figure S2.

## Discussion

### Patient-specific Calibration of Outflow Boundary Conditions

We presented a strategy for calibrating patient-specific outflow boundary conditions in the CoW. Our strategy relies on deriving mean flow splits in the main arteries of the CoW using ASL perfusion data, and on knowledge of the total flow to the head given by PC-MRI data in the neck arteries. The perfusion data in the different cerebral territories is the result of the spatial distribution of blood supply to the brain tissue, which is determined by the overall distal microvascular resistance and potentially cerebral auto-regulatory effects that seek to compensate deficits in flow in a region of the brain. Therefore, the calibrated outflow boundary condition parameters include the effect of all these mechanisms. Cerebral auto-regulatory compensation was apparent in patient 1, where the ASL-derived flow rates in the right and left MCA of the CoW were comparable despite a severe RICA stenosis. To match the ASL-derived mean flow rates in the CFD analysis, the distal resistance of each Windkessel model for the vessels in the CoW was iteratively adjusted during stage 1 of the calibration (cf. *Calibration of Windkessel model parameters*). The calibrated RMCA resistance was significantly lower than its LMCA counterpart. This finding points to a substantial vasodilation of the distal vasculature of the RMCA to maintain adequate blood supply to the brain tissue.

Previous CFD modeling studies of cerebral blood flow have relied primarily on assumptions on the flow distribution in the CoW, either based on literature data of healthy vasculatures (Xiao et al., 2013; Mukherjee et al., 2016) or allometric scaling laws (Bockman et al., 2012). However, in situations of cerebrovascular disease, the distribution of flow between the different vessels of the CoW may be substantially different from that given by idealized allometric scaling principles based on healthy data. Ultimately, incorrect values of flow in the vessels of the CoW will affect the quality of the CFD results. Zhang et al. (2016) previously presented a calibrated 1D-0D computational model of cerebral blood using single photon emission computed tomography (SPECT) to estimate the flow distribution in the CoW (Yamada et al., 2014). In this work, we built on this approach by acquiring non-invasive and non-radioactive NS-ASL perfusion images and using 3D models of blood flow, given by the incompressible Navier-Stokes equations, which are essential to capture complex hemodynamics around the stenosis and in the small and tortuous vessels of the CoW.

### Validation of Calibrated CFD Model

In this work, we demonstrated that our CFD calibration strategy of using NS-ASL perfusion images, in combination with a vascular territory atlas and PC-MRI, can accurately characterize flow in the main arteries of the CoW in a small group of subjects. The LPT analysis performed using the calibrated CFD model was validated using VS-ASL data by comparing their respective values of FBS in each vascular territory. Results showed an overall good agreement between LPT and VS-ASL with high correlation coefficients.

Bockman et al. (2012) developed a CFD model of the vertebrobasilar system with outflow boundary conditions defined *via* allometric scaling on healthy subjects without flow-altering CVOD. They studied the agreement in the laterality of the VA blood supply for the cerebral and cerebellar circulations between CFD and vessel-encoded ASL. ASL perfusion data was therefore not used to calibrate the outflow BC of the CFD model. Here, we modeled the entire CoW and validated blood supply to each vascular territory in both healthy and CVOD subjects. Furthermore, we demonstrated that the calibrated CFD models captured the collateral flow observed in the VS-ASL data.

### Assessment of Patient-Specific Cerebral Hemodynamics

Using the validated CFD model, we performed an in-depth quantification of cerebral hemodynamics in the two CVOD patients. Both patients presented with a severe RICA stenosis (70–99% diameter reduction) according to the velocity criteria (Patient 1), which correlates the peak systolic velocity measured with Duplex Ultrasound to a percentage diameter reduction, and the ECST criteria (Patient 2), which is defined as the diameter reduction relative to the original vessel diameter based on CTA. Despite similar degrees of clinical stenosis severity, the cerebral hemodynamics varied significantly between these two patients. In patient 1, the RICA stenosis led to severe ipsilateral pressure and flow drop compared to the contralateral unstenosed LICA. Despite the pressure difference between right and left hemisphere, there was no significant difference in flow between the right and left MCA due to collateral flow compensation and vasodilation of the distal vasculature. In contrast, the RICA stenosis in patient 2 did not result in a notable drop in ipsilateral pressure or flow. Consequently, the flow compensation between hemispheres was small. The difference in flow compensation between the two patients is explained by the much smaller stenosis diameter of patient 1 (1.4 mm in our geometric model, 74.7% diameter reduction relative to the distal diameter) compared to patient 2 (3.0 mm, 52.3% diameter reduction relative to distal diameter), see Figure 1. Flow compensation is highly dependent on accurate characterizations of the degree of stenosis, the cerebral anatomy, and the cerebrovascular reserve and can vary significantly between patients.

Medical imaging used for cerebral hemodynamics assessment (e.g. Transcranial Doppler, 4D Flow MRI, etc.) only provide information on velocity. However, the above results (similar cerebral flow between the two subjects while having substantial differences in pressure) highlight the shortcomings of describing the hemodynamic significance of CVOD lesions purely from the perspective of velocity. In contrast, blood pressure is highly sensitive to changes in vascular resistance induced by the stenosis, as illustrated in patient 1. Therefore, substantial changes in cerebral blood pressure may be a more sensitive marker for diminished vascular flow reserve. While pressure catheter measurements are commonly acquired in other vascular territories (e.g. coronary arteries), cerebral blood pressure is generally not acquired during clinical assessment of CVOD patients due to increased stroke risk. While there have been efforts to derive pressure gradients from 4D Flow MRI data, application in the small and tortuous CoW arteries remains challenging due to limited spatial resolution (Vali et al., 2019). In contrast, patient-specific CFD overcomes these shortcomings by providing highly resolved velocity and pressure.

To quantify the stenosis hemodynamic significance, we calculated the fractional flow index, defined as the ratio of the pressures distal and proximal to the stenosis under baseline flow conditions (e.g., non-hyperemic). The fractional flow in the RICA stenosis was FF_*RICA*_ = 0.71 for patient 1 and FF_RICA_ = 0.98 for patient 2. Using a threshold of FF = 0.8 (Miao et al., 2016), only the RICA stenosis in patient 1 would be deemed to be hemodynamically significant. While the clinical metric of diameter reduction resulted in a similar value of 70–99% in the RICA for both patients, the fractional flow index captured better the large differences in cerebral hemodynamics between the patients. The metric of fractional flow reserve (FFR) has become widely used in coronary artery disease (CAD) to evaluate the risk for myocardial ischemia. FFR-guided intervention has been shown to reduce myocardial ischemia, rate of death, and revascularization compared to anatomical-based intervention (Tonino et al., 2009; De Bruyne et al., 2014). However, the use of fractional flow for the risk assessment of ischemic stroke in CVOD has not yet been established.

In this work, the CFD analysis focused primarily on assessing the hemodynamic effects of carotid stenosis on flow and pressure, as well as the cerebral vasculature’s capacity to compensate flow. However, it is important to note that the formation of emboli from the carotid plaque is thought to be the major mechanism of ischemic stroke in patients with carotid stenosis, especially in asymptomatic patients (King et al., 2011). The assessment of plaque composition, in particular the size of the lipid-rich necrotic core and the fibrous cap, is key to determining plaque vulnerability and embolic stroke risk. However, hemodynamic factors (e.g. wall shear stress and blood pressure) are suspected to also play an important role in plaque vulnerability (Chen et al., 2020).

FBS obtained from CFD and LPT provided important information about collateral flow in the anterior circulation and mixed VA supply in the posterior circulation. Beyond flow compensation, FBS in the cerebral vascular territories can provide clinically relevant information about the etiology of embolic stroke. Even after thorough diagnostic evaluation, the cause of embolic stroke remains uncertain in one third of cases (Nouh et al., 2016). It is common that multiple atherosclerotic lesions within the same patient are identified. Evaluation of FBS in the region of the stroke could help to determine the source of emboli and ultimately guide clinical treatment.

These results illustrate the level of insight on hemodynamic assessment of CVOD patients that calibrated patient-specific CFD analysis can bring.

### Limitations

#### Vascular Territory Atlas

In this work, the flow splits between the main intracranial arteries were estimated from non-selective ASL perfusion images. The segmentation of the perfusion images relied on knowledge of the vascular territories corresponding to the intracranial arteries. Since the precise location of the territories was not available, which is generally the case, a vascular territory atlas was used to define the boundaries of each territory. Vascular territory atlases are routinely used in the radiologic assessment of infarctions to investigate the stroke mechanism and to guide further therapy (Phan et al., 2005). A major limitation of most available atlases (Tatu et al., 2012) is the small sample size of subjects used to derive territory maps, resulting in large uncertainties in the border zones. The atlas used in this work was recently developed by Kim et al. based on a large population study of 1,160 stroke patients with intracranial stenosis (Kim et al., 2018). In their study, the authors showed that the border zones of the derived atlas were much narrower than previously assumed, which justified assigning territory boundaries based on the probability of each image voxel of being associated with each intracranial artery. However, some degree of variability in the vascular territories is expected among patients. For example, the formation of leptomeningeal collaterals between the distal branches of the CoW can lead to changes in the vascular territories (Tariq and Khatri, 2008). In the presence of patent leptomeningeal collaterals, the additional pathways would have to be accounted for in the geometric model and in the segmentation of the territories.

#### VS-ASL

The acquisition of VS-ASL images is generally challenging due to the inherently low signal-to-noise ratio. Cardiac pulsatility, B_0_-field inhomogeneity, and subject movement can lead to further artifacts in the territorial perfusion maps (Schollenberger et al., 2020). For example, signal fluctuations between label and control images can result in spurious perfusion signal in vascular territories not perfused by the labeled neck artery. Previous applications of VS-ASL have been limited to a qualitative description of territorial perfusion. In this work, we quantified VS-ASL image data by deriving the fractional blood supply. To account for the spatial variability of FBS within a vascular territory due to noise and image artifacts, we included the median absolute deviation in the data as shown in Figure 5.

#### PC-MRI

Estimation of mean flow rates in the intracranial arteries relies on knowledge of the total flow to the CoW. However, PC-MRI flow measurements in the neck arteries might not always be available. As an alternative approach, intracranial flow rates could be estimated exclusively from the non-selective ASL data. In this work, we quantified the ASL image data by calculating the averaged signal difference between control and label images. While the signal difference is proportional to perfusion, the units are arbitrary. However, one of the advantages of ASL is the ability to quantify the difference images in units of perfusion (e.g. ml/min/100 g). This can be achieved by using for example a single compartment model (Buxton et al., 1998). Once the perfusion images are quantified, mean intracranial flow rates can be derived by integrating perfusion over each segmented territory using the vascular territory atlas and multiplying with the brain tissue density. While the resulting total flow to the CoW might be different from the one measured with PC-MRI, the ratio of flow rates across the intracranial arteries and consequently the collateral flow would be maintained.

## Conclusion

In this work, we presented a strategy to quantify cerebral hemodynamics using CFD in combination with ASL and PC-MRI data. We demonstrated that our calibrated CFD model accurately reproduced the fractional blood supply to the vascular territories, as obtained from VS-ASL. In particular, the flow compensation between hemispheres was captured well by the calibrated CFD models. The assessment of cerebral hemodynamics in two CVOD patients using calibrated patient-specific CFD analysis showed significant differences in cerebral hemodynamics between the patients despite similar degrees of clinical stenosis severity. We further illustrated the advantages of CFD-based pressure data for assessing the hemodynamic significance of carotid stenosis. Future studies are needed to investigate the benefits of using of a hemodynamic-based metric (fractional flow) versus an anatomy-based metric (diameter reduction) for the risk assessment of CVOD patients.

## Data Availability

The raw data supporting the conclusions of this article will be made available by the authors, without undue reservation.
