# Supplementary material for: A Combined Computational Fluid Dynamics and Arterial Spin Labeling MRI Modeling Strategy to Quantify Patient-Specific Cerebral Hemodynamics in Cerebrovascular Occlusive Disease
Source: Front Bioeng Biotechnol. 2021 Aug 17;9:722445. doi: 10.3389/fbioe.2021.722445 (PMC8416094; doi:10.3389/fbioe.2021.722445)
Supplement: Supplementary file 2 [file DataSheet2.docx]

Supplementary Material

# Supplementary Figures


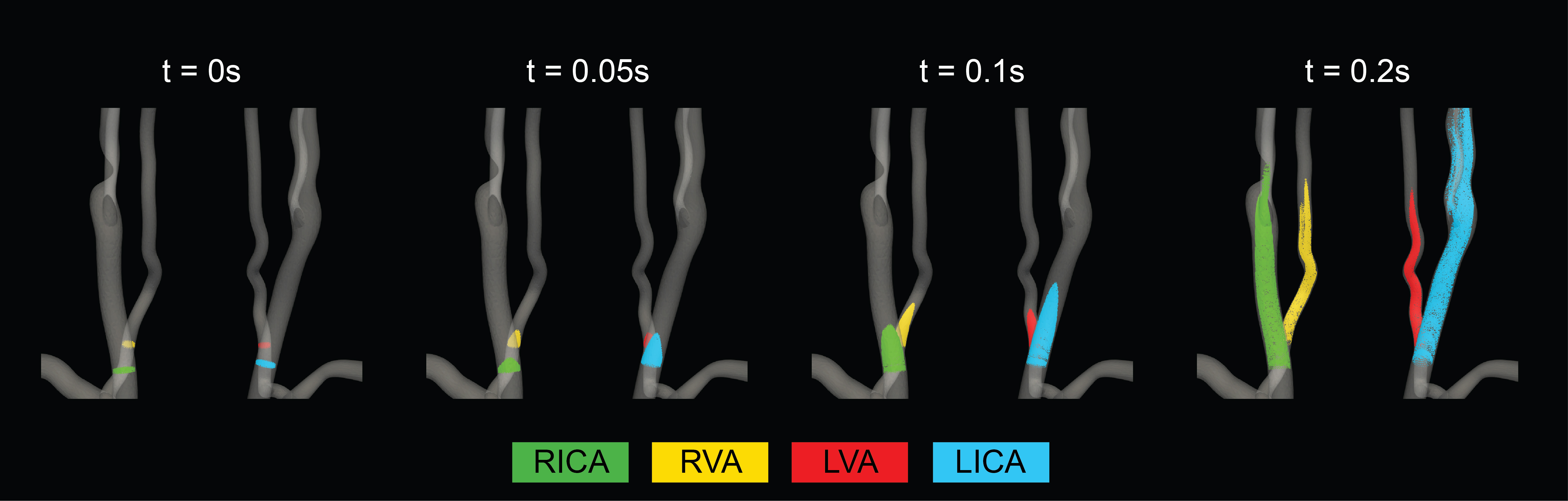


**Fig. S1:** Continuous seeding and advection of Lagrangian particles in the vertebral and carotid arteries at four time points.


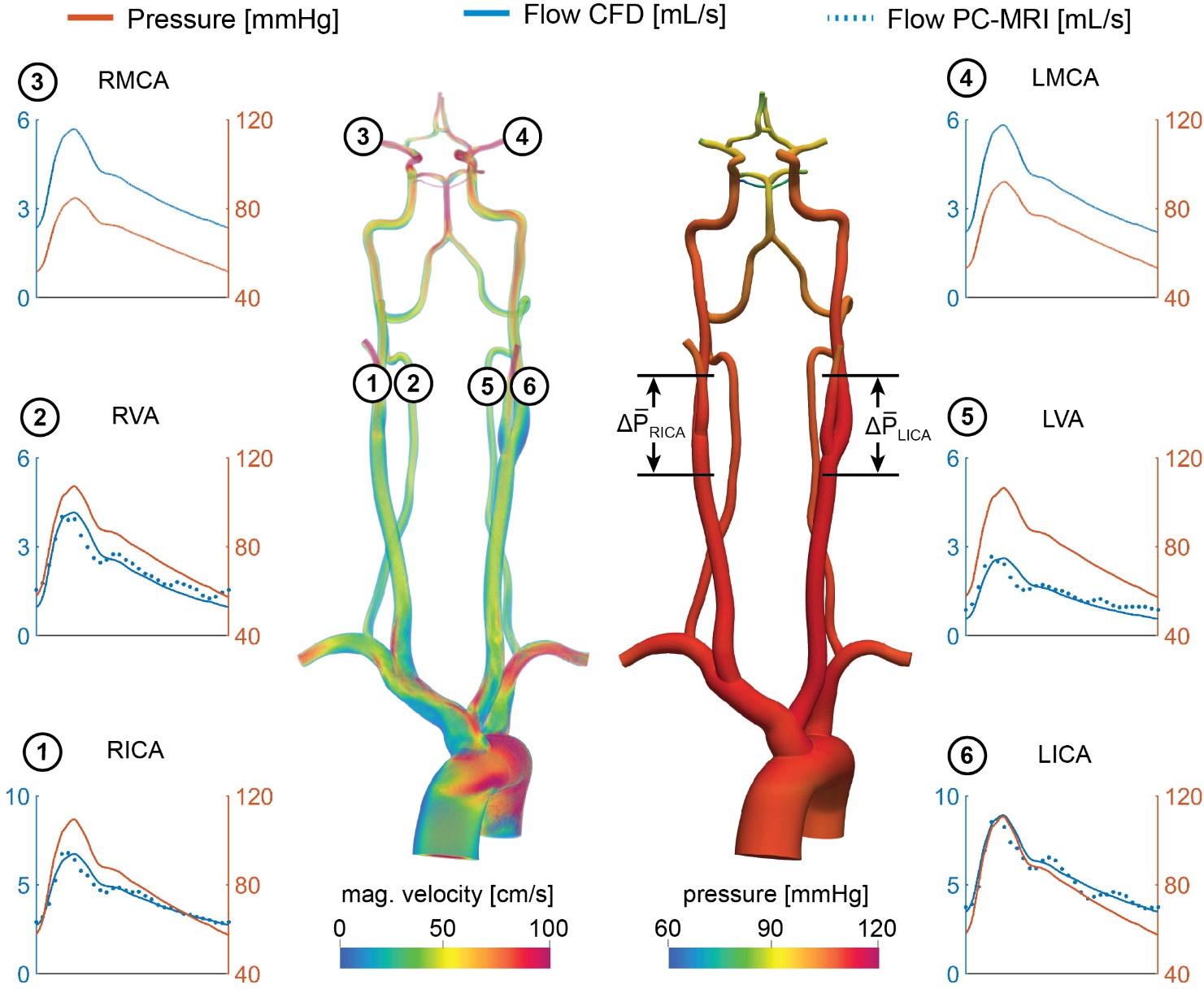


**Fig. S2:** Velocity and pressure fields at peak systole are shown for the healthy control subject. Flow and pressure waveforms are evaluated in the internal carotid (ICA), vertebral (VA), and middle cerebral arteries (MCA) and compared to PC-MRI measurements in the neck arteries. A pressure drop and fractional flow over the patent right and left carotid bifurcations were calculated analog to the patient examples for reference, yielding $\Delta\bar{P}_{RICA}=0.77 mmHg$ and $\Delta\bar{P}_{LICA}=0.55 mmHg$ and correspondingly ${FF}_{RICA}=0.99$and ${FF}_{LICA}=0.99$. The flow rates in the RMCA and LMCA were similar with $\bar{Q}_{RMCA}=3.71 mL/s$ and $\bar{Q}_{LMCA}=3.62 mL/s$ and the pressure difference between these outlets was $\Delta\bar{P}_{MCA}=3.61 mmHg$.

# Supplementary tables


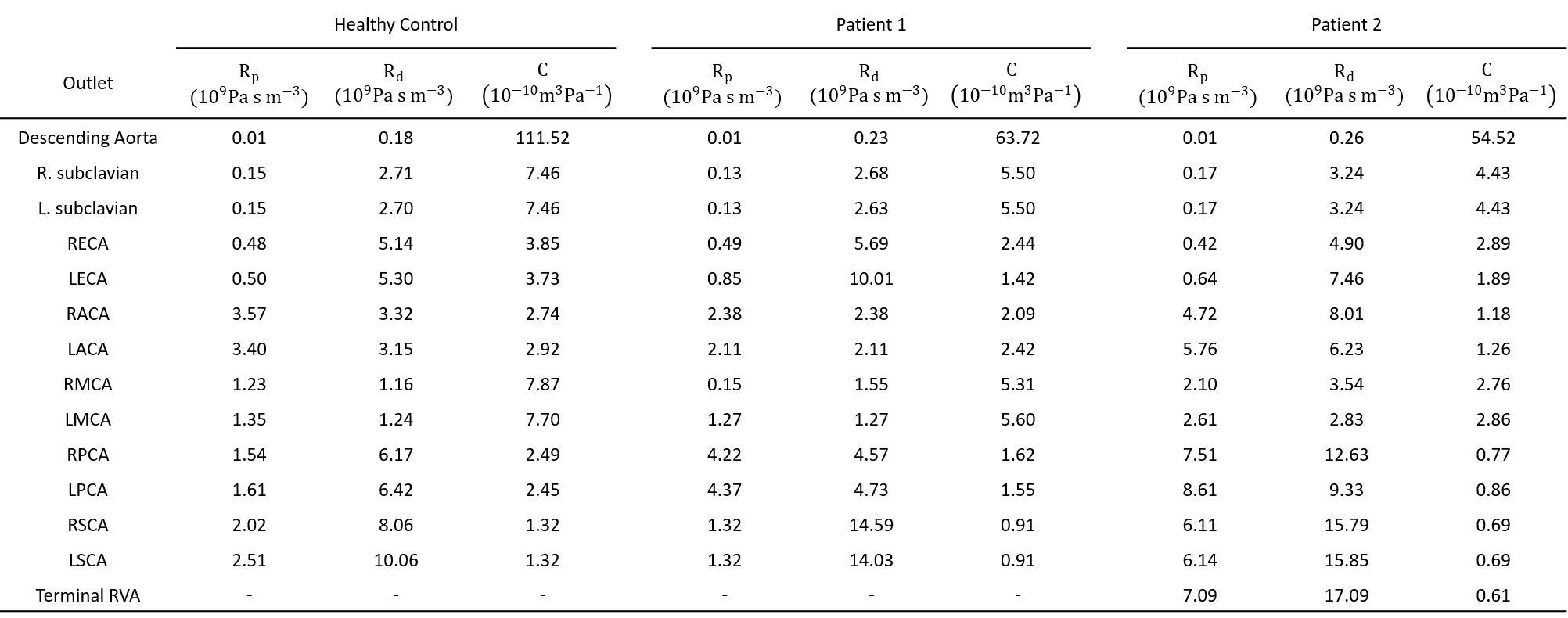


**Table S1:** Parameters of the calibrated 3-element Windkessel models for all study subjects. Each Windkessel model consists of a proximal resistance $R_{p}$, distal resistance $R_{d}$, and compliance $C$. RECA/LECA = right/left external carotid artery, RACA/LACA = right/left anterior cerebral artery, RMCA/LMCA = right/left middle cerebral artery, RPCA/LPCA = right/left posterior cerebral artery, RSCA/LSCA = right/left superior cerebellar artery, RVA = right vertebral artery.
